# Pericardio-peritoneal communication following emergency subxiphoid pericardiocentesis in acute complex percutaneous coronary intervention: a case report

**DOI:** 10.1093/ehjcr/ytag702

**Published:** 2026-09-26

**Authors:** Tharusan Thevathasan, Ulf Landmesser, Arbnora Magdalena Apling, Carsten Skurk, Youssef Abdelwahed

**Affiliations:** Department of Cardiology, Angiology and Intensive Care Medicine, Deutsches Herzzentrum der Charité (DHZC), Campus Benjamin Franklin, Hindenburgdamm 30, 12203 Berlin, Germany; Berlin Institute of Health, Anna-Louisa-Karsch-Straße 2, 10178 Berlin, Germany; Deutsches Zentrum für Herz-Kreislauf-Forschung, Partner Seite Berlin, Potsdamer Str. 58, 10785 Berlin, Germany; Department of Cardiology, Angiology and Intensive Care Medicine, Deutsches Herzzentrum der Charité (DHZC), Campus Benjamin Franklin, Hindenburgdamm 30, 12203 Berlin, Germany; Berlin Institute of Health, Anna-Louisa-Karsch-Straße 2, 10178 Berlin, Germany; Deutsches Zentrum für Herz-Kreislauf-Forschung, Partner Seite Berlin, Potsdamer Str. 58, 10785 Berlin, Germany; Department of Cardiology, Angiology and Intensive Care Medicine, Deutsches Herzzentrum der Charité (DHZC), Campus Benjamin Franklin, Hindenburgdamm 30, 12203 Berlin, Germany; Department of Cardiology, Angiology and Intensive Care Medicine, Deutsches Herzzentrum der Charité (DHZC), Campus Benjamin Franklin, Hindenburgdamm 30, 12203 Berlin, Germany; Deutsches Zentrum für Herz-Kreislauf-Forschung, Partner Seite Berlin, Potsdamer Str. 58, 10785 Berlin, Germany; Department of Cardiology, Angiology and Intensive Care Medicine, Deutsches Herzzentrum der Charité (DHZC), Campus Benjamin Franklin, Hindenburgdamm 30, 12203 Berlin, Germany

**Keywords:** Pericardiocentesis, Cardiac tamponade, Pericardial drainage, Complications, Pericardio-peritoneal communication, Complex PCI, Covered stent, Case report

## Abstract

**Background:**

Emergency pericardiocentesis represents a life-saving intervention in patients with acute cardiac tamponade. During complex percutaneous coronary intervention (PCI), abrupt haemodynamic deterioration secondary to pericardial tamponade may necessitate immediate subxiphoid drainage. Although the procedure is generally safe when performed by experienced operators, catheter malposition can result in ineffective pericardial decompression and may be associated with unexpected and potentially serious complications.

**Case summary:**

An 83-year-old patient with multiple cardiovascular risk factors and paroxysmal atrial fibrillation underwent high-risk PCI for severely calcified left main and multivessel coronary artery disease at an external institution. Owing to persistent angina, the patient was transferred directly to our tertiary referral centre. Coronary angiography revealed an iatrogenic left main coronary artery dissection, presumably related to the preceding PCI. The lesion was successfully treated using intravascular imaging guidance, adjunctive plaque modification, and drug-eluting stent implantation, resulting in complete sealing of the dissection and restoration of Thrombolysis in Myocardial Infarction 3 flow. Shortly thereafter, the patient developed acute hypotension accompanied by echocardiographic evidence of rapidly accumulating pericardial effusion with haemodynamic features of cardiac tamponade. Emergency subxiphoid pericardiocentesis led to immediate haemodynamic stabilization. However, persistent abdominal discomfort prompted further diagnostic evaluation. Computed tomography (CT) demonstrated intraperitoneal contrast distribution across multiple abdominal quadrants, consistent with peritoneal perforation by the pigtail catheter and the presence of an iatrogenic pericardio-peritoneal communication. Serial transthoracic echocardiography showed only minimal and stable residual pericardial effusion without recurrence of tamponade physiology. The drainage catheter was therefore removed at an early stage. Follow-up CT confirmed progressive resolution of intraperitoneal contrast. The patient subsequently recovered without further complications.

**Discussion:**

Peritoneal perforation during subxiphoid pericardiocentesis is a rare but clinically relevant complication. In cases in which clinical symptoms are disproportionate to echocardiographic findings, or when the effectiveness of pericardial drainage is uncertain, early cross-sectional imaging should be considered to verify catheter position and to identify abnormal extracardiac fluid communication. Prompt recognition and timely catheter removal may prevent further complications and obviate the need for surgical intervention.

Learning points

- A steep subxiphoid needle trajectory during emergency pericardiocentesis may cause peritoneal perforation and iatrogenic pericardio-peritoneal communication.
- Persistent abdominal symptoms or discordant imaging findings after pericardiocentesis should prompt early CT to verify catheter position and detect extracardiac contrast or fluid distribution.
- In haemodynamically stable patients without recurrent tamponade, early catheter removal with serial clinical and echocardiographic monitoring may allow conservative resolution.

## Introduction

High-risk percutaneous coronary intervention (PCI) in the setting of complex, calcified left main and multivessel coronary artery disease may be complicated by coronary dissection and periprocedural bleeding, necessitating immediate bail-out strategies. Peri-interventional pericardial tamponade remains a time-critical emergency in the catheterization laboratory and demands prompt diagnosis and decompression.^1^ Subxiphoid pericardiocentesis is widely used due to its favourable access route and familiarity among interventional teams. Nevertheless, catheter malposition and extra-pericardial drainage can occur and may create diagnostic uncertainty when clinical presentation is atypical or when drainage efficacy is questioned.^2^ We report an iatrogenic pericardio-peritoneal communication caused by peritoneal perforation during emergency subxiphoid pericardiocentesis following complex left main PCI, emphasizing imaging-based confirmation and a conservative, catheter-focused management strategy.

## Summary figure

**Figure:**
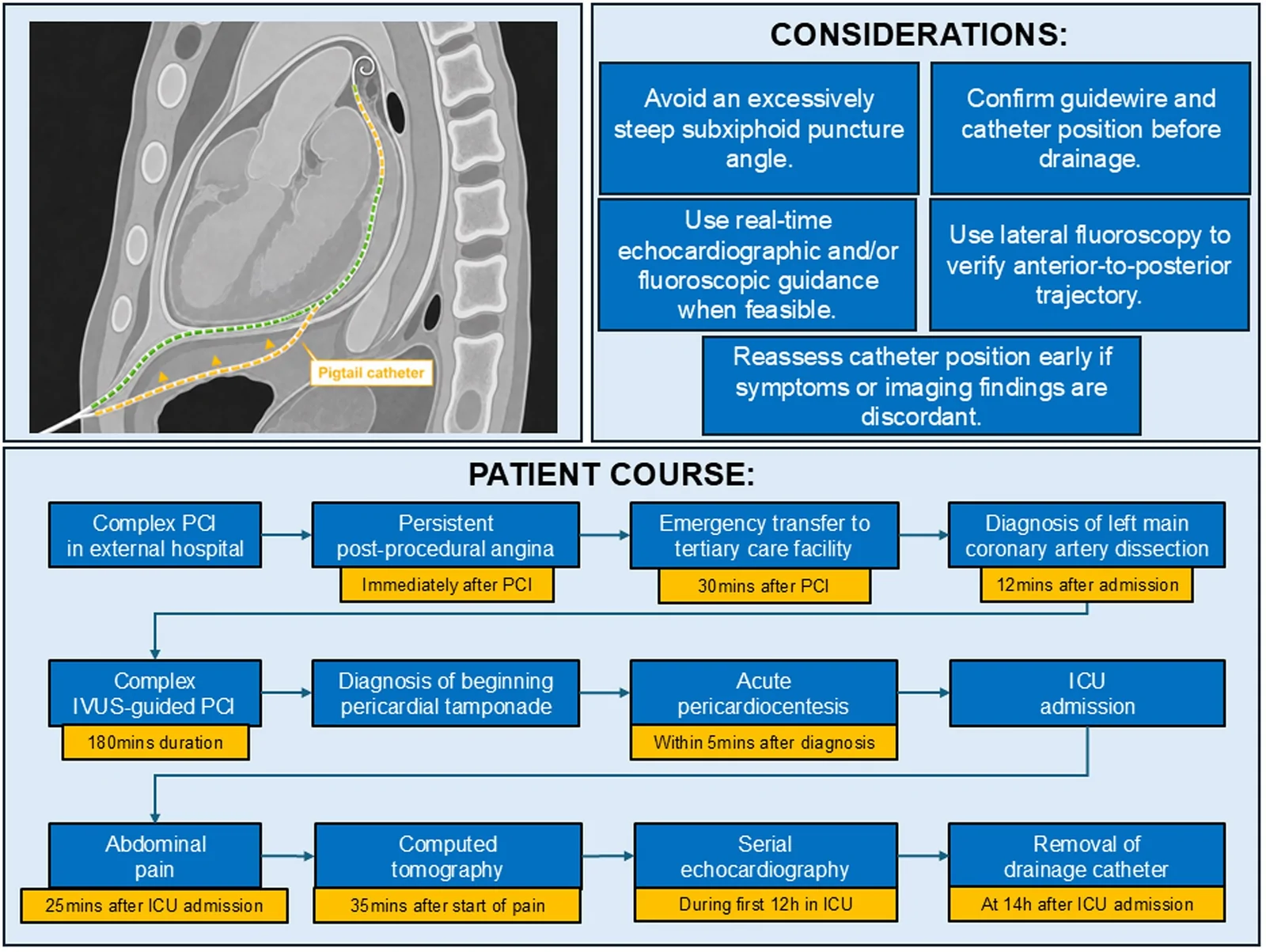
The left upper panel shows subxiphoid pigtail catheter placement with an excessively steep puncture trajectory (orange course), resulting in passage through the peritoneal cavity into the pericardial space and thereby creating a pericardio-peritoneal communication. The less steep trajectory (green course) illustrates the intended subxiphoid access route to the pericardial space, avoiding peritoneal perforation and fistula formation. The right upper panel summarizes key procedural considerations. The lower panel summarizes the clinical course of the patient.

## Case presentation

An 83-year-old patient with a history of dyslipidaemia, arterial hypertension, previous tobacco use, paroxysmal atrial fibrillation, and prostate cancer presented to an external hospital with exertional angina. Physical examination, resting electrocardiography, and routine laboratory testing were unremarkable. Baseline haemoglobin was 12.7 g/dL and serum creatinine was 1.24 mg/dL with an estimated glomerular filtration rate 58 mL/min/1.73 m^2^. Transthoracic echocardiography demonstrated preserved left ventricular systolic function with regional anterior wall motion abnormalities. Invasive coronary angiography revealed severe three-vessel coronary artery disease with heavily calcified stenoses involving the left main coronary artery and the left anterior descending (LAD) artery.

Percutaneous coronary intervention of the proximal LAD was performed at the referring centre including loading with aspirin only during the procedure and clopidogrel after the procedure, while baseline anticoagulation with apixaban for atrial fibrillation had been established before admission. Owing to persistent angina, the patient was immediately transferred to our tertiary care centre for further management. According to the procedural report from the referring hospital, no procedural complications were documented during the initial PCI. On arrival, the patient remained haemodynamically stable and was scheduled for an urgent repeat coronary intervention. Coronary angiography demonstrated a dissection extending from the distal left main coronary artery into the LAD, presumed to be iatrogenic and related to the preceding PCI in the proximal LAD at the referring hospital (see Supplementary material online, *Video S1*). At the time of repeat angiography, the patient had ongoing angina and a left main/LAD lesion with angiographic features suggesting an unstable dissection. Given the acute clinical presentation, the need for immediate restoration and preservation of coronary flow, and the expected time delay associated with emergency surgical revascularization, the treating interventional team proceeded with urgent intravascular ultrasound (IVUS)–guided PCI rather than deferring for surgical treatment.

Intravascular ultrasound was used to characterize lesion morphology and to guide the interventional strategy (*Figure 1*). Given the marked coronary calcification, adjunctive intravascular lithotripsy was required. To restore vessel integrity, multiple drug-eluting stents were implanted from the left main coronary artery into the LAD, resulting in complete sealing of the dissection flap.

**Figure 1:**
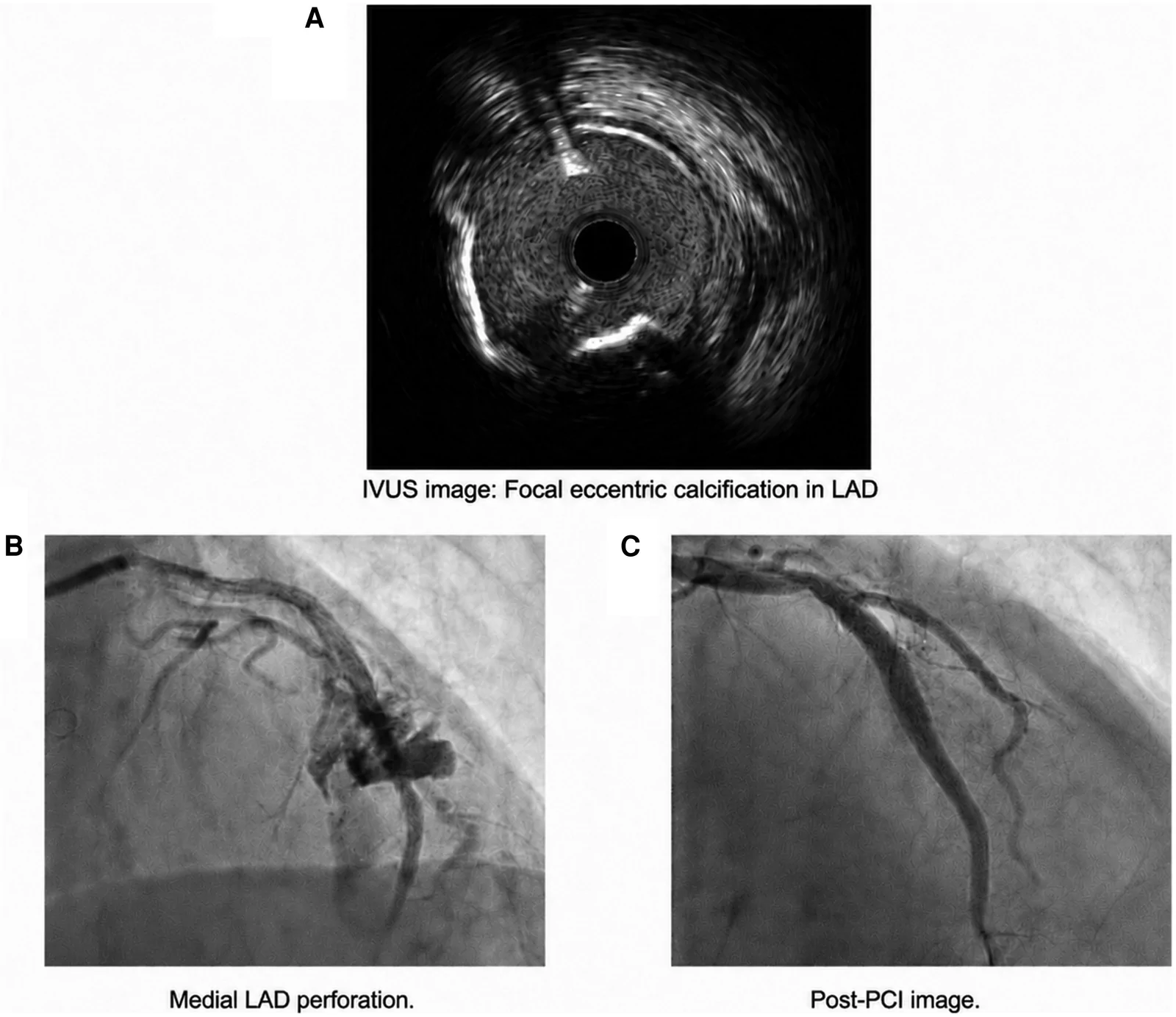
Coronary angiography and intravascular ultrasound-guided percutaneous coronary intervention of the left anterior descending artery. (*A*) shows IVUS demonstrating focal eccentric calcification in the LAD. (*B*) demonstrates medial LAD perforation during the intervention with contrast extravasation. (*C*) shows the final post-PCI angiographic result after treatment, with restoration of vessel patency and no persistent major contrast extravasation. IVUS, intravascular ultrasound; LAD, left anterior descending artery; PCI, percutaneous coronary intervention.

Shortly thereafter, the patient developed acute hypotension. Transthoracic echocardiography demonstrated a rapidly accumulating pericardial effusion with haemodynamic features consistent with evolving cardiac tamponade. Coronary angiography demonstrated perforation of the mid-LAD (*Figure 1*). The rapidly accumulating pericardial effusion was considered procedure-related, occurring after massive debulking and lesion preparation. Emergency pericardiocentesis via a subxiphoid approach was immediately performed under combined echocardiographic and fluoroscopic guidance using anteroposterior projection, leading to prompt haemodynamic stabilization. The needle was advanced with an intended anterior subxiphoid trajectory towards the pericardial space. Catheter position was assessed by fluoroscopy as well as aspiration of 820 mL haemorrhagic fluid, supporting the diagnosis of procedure-related haemopericardium. The mid-LAD was immediately treated with covered stent implantation and the subsequent haemodynamic improvement after pericardial drainage was interpreted as evidence of effective pericardial decompression. Haemoglobin decreased from 12.7 g/dL before the intervention to 8.6 g/dL after pericardiocentesis. The patient received three units of packed red blood cells, after which haemoglobin was stabilized around 14 g/dL. Procedural risk factors for pericardiocentesis included emergency catheter laboratory admission, complex PCI, antithrombotic and anticoagulant exposure, and the urgent need for rapid tamponade decompression.

The coronary procedure was subsequently completed with restoration of Thrombolysis in Myocardial Infarction 3 flow and preserved myocardial perfusion (*Figure 2*). Mechanical circulatory support was considered before the start of the PCI and kept on standby for immediate use if required; however, it was not implanted upfront because the patient was haemodynamically stable at the start of the procedure, coronary flow was preserved, and the anticipated intervention was directed at sealing the dissection and perforation rather than treating established cardiogenic shock. Since haemodynamic deterioration was caused by acute cardiac tamponade, the immediate therapeutic priority was pericardial decompression, after which mechanical circulatory support was ultimately not required.

**Figure 2:**
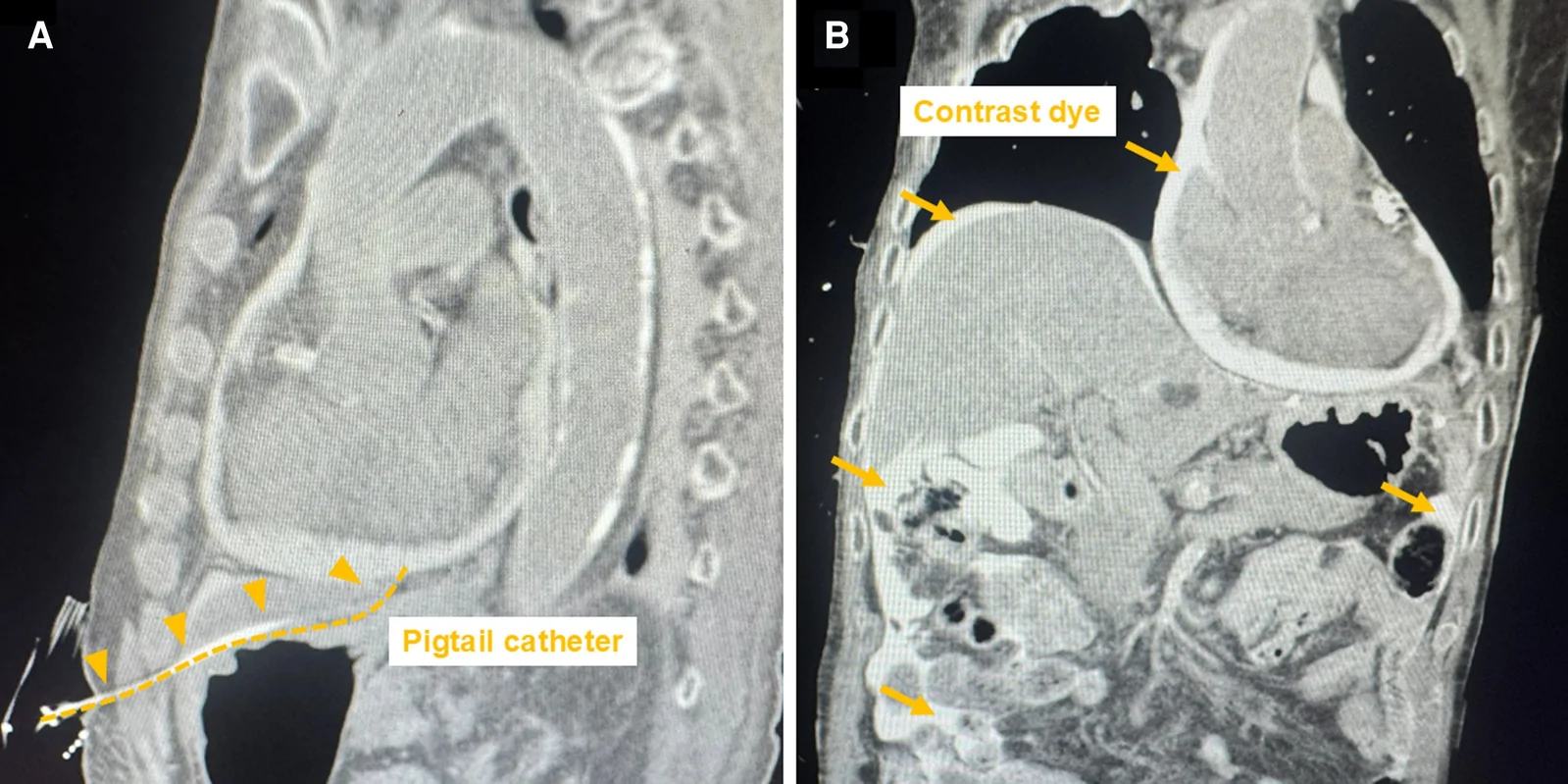
CT imaging demonstrating iatrogenic pericardio-peritoneal communication. (*A*) Sagittal reconstruction showing the course of the pericardial pigtail catheter following subxiphoid drainage, with transperitoneal catheter passage consistent with peritoneal perforation. The arrowheads mark the course of the pigtail catheter across the peritoneal cavity. (*B*) Coronal reconstruction demonstrating intraperitoneal distribution of contrast dye across multiple abdominal quadrants (arrows), consistent with peritoneal perforation and pericardio-peritoneal communication. The intraperitoneal contrast was attributed to contrast-containing pericardial fluid entering the abdominal cavity through the catheter-related pericardio-peritoneal communication after contrast administration during complex percutaneous coronary intervention and pericardial drainage. Arrows indicate intraperitoneal contrast distribution. CT, computed tomography.

Because of persistent abdominal discomfort following the intervention, post-procedural computed tomography (CT) was performed. Cross-sectional imaging demonstrated contrast medium distributed throughout the peritoneal cavity across multiple abdominal quadrants. The pericardial drainage catheter was visualized traversing the peritoneum, consistent with iatrogenic peritoneal perforation and the formation of a pericardio-peritoneal communication. The catheter tip was located within the pericardial space. Importantly, CT demonstrated no evidence of active arterial extravasation, liver injury, bowel injury, gastric perforation, or expanding intra-abdominal haematoma. General surgical consultation was obtained and conservative management was supported because there were no clinical signs of peritonitis and no CT evidence of intra-abdominal organ injury. Antibiotic prophylaxis with piperacillin/tazobactam was administered. Therapeutic oral anticoagulation with apixaban was temporarily withheld because of the acute bleeding complication, and pharmacological thromboprophylaxis was administered during the initial post-procedural period. Despite the bleeding complication, dual antiplatelet therapy with aspirin and clopidogrel was continued because of the very recent complex left main/LAD drug-eluting stent implantation.

Serial transthoracic echocardiography showed only minimal and stable residual pericardial effusion without recurrence of tamponade physiology. In the absence of ongoing haemodynamic compromise, the drainage catheter was removed at an early stage. Total drainage volume during the total 14 h was 1280 mL. Repeat CT confirmed progressive resolution of intraperitoneal contrast. Because there was no clinical, laboratory, or radiological evidence of ongoing intra-abdominal haemorrhage, repeat invasive investigation was not pursued. Therapeutic anticoagulation with apixaban was resumed after serial echocardiography and repeat CT had excluded ongoing pericardial or intra-abdominal bleeding. The patient was monitored for 12 additional hours in an intensive care setting with serial clinical examinations, repeated haemoglobin measurements, haemodynamic monitoring, and serial transthoracic echocardiography to exclude recurrent tamponade. The patient recovered uneventfully and was discharged without further complications. At discharge, aspirin was discontinued and the patient was maintained on clopidogrel and apixaban.

## Discussion

This case illustrates an uncommon complication of emergency pericardiocentesis, namely, peritoneal perforation with the formation of an iatrogenic pericardio-peritoneal communication. Although the subxiphoid approach is frequently preferred in emergency settings, it traverses anatomical planes in close proximity to the diaphragm, liver, and peritoneal cavity.^3^ A steep needle trajectory, limited imaging guidance, or altered anatomical conditions may therefore increase the likelihood of inadvertent peritoneal entry, resulting in extra-pericardial catheter placement and potentially ineffective or misleading drainage.^4,5^

In the present case, initial haemodynamic improvement after pericardial drainage created a degree of false reassurance. However, the persistence of abdominal symptoms prompted cross-sectional imaging, which revealed intraperitoneal dissemination of contrast material. This sequence highlights a critical practical principle: when post-procedural symptoms are atypical, particularly abdominal discomfort, or when the clinical course appears discordant with the echocardiographic evolution of pericardial effusion, verification of catheter position should not be delayed. While echocardiography remains the cornerstone for the diagnosis of cardiac tamponade and for guidance of pericardial access, cross-sectional imaging is indispensable when extra-thoracic complications are suspected.

A pericardio-peritoneal communication may function as an unintended drainage pathway for contrast medium and pericardial effusion, thereby obscuring recurrent tamponade or complicating the interpretation of ongoing bleeding. In the context of recent complex coronary intervention and treatment of coronary dissection, precise discrimination between intra-pericardial and extra-pericardial fluid distribution is essential, as management strategies differ fundamentally, ranging from continued pericardial drainage and haemostatic measures to catheter repositioning or removal and clinical observation.

In the present patient, serial echocardiography demonstrated only minimal and stable residual pericardial effusion and haemodynamic stability was maintained. Under these conditions, early catheter removal was justified and resulted in radiographic resolution without the need for surgical intervention. Importantly, a conservative approach is only defensible when all of the following criteria are met: absence of recurrent or ongoing tamponade, stability or regression of pericardial effusion on serial imaging, lack of clinical or radiological evidence of peritonitis or intra-abdominal bleeding, and consensus within a multidisciplinary team supporting non-operative management.

Preventive strategies are pragmatic and procedural—rather than technically complex. They include deliberate control of needle trajectory with avoidance of excessive angulation, systematic use of imaging guidance whenever feasible, particularly real-time echocardiography and/or fluoroscopy and immediate confirmation of catheter position when haemodynamic response is incomplete or atypical.^6^ A pure lateral fluoroscopic projection can be particularly useful to select and confirm the anterior-to-posterior needle angle during subxiphoid pericardiocentesis. This may be especially relevant in overweight patients, in patients with distorted thoracoabdominal anatomy or when echocardiographic windows are limited. In retrospect, the absence of systematic lateral fluoroscopic confirmation may have contributed to failure to recognize the excessively steep trajectory before catheter placement in this case. Clinicians should maintain a low threshold for early CT when abdominal symptoms develop after a subxiphoid puncture or when contrast distribution appears unexpected.

In summary, peritoneal perforation during subxiphoid pericardiocentesis is a rare but clinically significant complication that can compromise effective management of cardiac tamponade. Early recognition through cross-sectional imaging and prompt, catheter-directed management allow safe resolution in selected patients and may prevent escalation to surgical intervention.

## Lead author biography


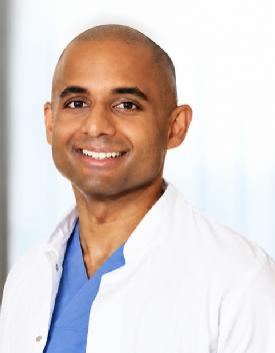
Dr Tharusan Thevathasan is a physician in the Department of Cardiology, Angiology and Intensive Care Medicine and a research coordinator at the Berlin Cardiac Arrest Center of the Deutsches Herzzentrum der Charité (Campus Benjamin Franklin). His clinical and academic focus is on interventional cardiology, cardiogenic shock, refractory cardiac arrest, and mechanical circulatory support. Dr Thevathasan has conducted research in critical care medicine at leading institutions including Massachusetts General Hospital/Harvard Medical School, University College London, and Oxford University Hospitals. He is dedicated to advancing patient-centred care and interdisciplinary research in acute cardiovascular emergencies.

## Supplementary Material

ytag702_Supplementary_Data

## Data Availability

All relevant clinical data are presented within the article. Further anonymized data can be made available upon reasonable request to the corresponding author.
